# Application of dual targeting drug delivery system for the improvement of anti-glioma efficacy of doxorubicin

**DOI:** 10.18632/oncotarget.19221

**Published:** 2017-07-13

**Authors:** Zhenliang Sun, Xuebing Yan, YiBo Liu, Linsheng Huang, Cheng Kong, Xiao Qu, Man Wang, Renyuan Gao, Huanlong Qin

**Affiliations:** ^1^ Department of General Surgery, Shanghai Tenth People's Hospital Affiliated to Tongji University, Shanghai 200072, China; ^2^ Shanghai Jiaotong University Affiliated Sixth People's Hospital, South Campus, Shanghai 201499, China; ^3^ Longhua Hospital of Shanghai University of TCM, Shanghai 200032, China

**Keywords:** tumor affinity peptide, interleukin-4 receptor, solid tumors, anti-glioma, nanoparticle

## Abstract

Chemotherapy of glioma is always hampered by the unsatisfactory tumor accumulation of drugs, of which the most noticeable obstacle is the limited drug permeability from vessels into tumor inner. In the present study, we developed a novel nanocarrier for the delivery of doxorubicin to brain tumor. Such novel drug delivery system was mainly composed of a tumor homing peptide and DOX-loaded PLA nanoparticles (AP1-NP-DOX). CRKRLDRNC peptide, named as AP1, was a newly glioma affinity peptide which could specifically binds to interleukin-4 receptor (IL-4R), highly expressing on both glioma cells and angiogenesis. Our findings showed that the peptide-functionalized nanoparticles had a high affinity with both tumor cells and vascular endothelial cells. Besides, tumor targeting assay exhibited that AP1 decorated nanoparticles accumulated more in tumor site than the unmodified ones. Moreover, the results of tumor uptake experiments indicated that AP1-NP-DOX might own the ability of blood brain barrier (BBB) penetration. In the anti-glioma study, AP1-NP-DOX exhibited the highest therapeutic effect on tumor-bearing mice compared with the unmodified nanoparticles and free doxorubicin. These results together indicated that AP1-functionalized nanoparticles could represent a promising way to expand the treatment horizons of onco-therapy.

## INTRODUCTION

Glioma, highly resistant to radiotherapy and most chemotherapy, represents one of the most aggressive brain tumors, with a very low survival rate of 5% for 5 years [1–3]. Conventional surgical methods and chemotherapy comprise the primary clinical treatment strategies. However, as a central nervous system (CNS) tumor, surgical resection comes with enormous difficulties. Furthermore, the nonspecific, nontargeted properties of most of the currently used drugs such as paclitaxel, doxorubicin and hydroxycamptothecin always lead to a limited accumulation of active agents in tumor and low drug permeability from vessels into brain tumor cells [4]. Therefore, the comprehensive effect of chemotherapy is always challenged and a higher accumulation of agents at specific tumor sites was the prerequisite of success tumor treatment by chemotherapeutics [5]. Fortunately, many major new technologies related to drug delivery have changed the scientific landscape of tumor targeting delivery of chemotherapeutics over the past few decades [6].

Those developed strategies including nanoparticle-based cancer therapies, transdermal patches, oral drug delivery, pulmonary drug delivery, implantable systems and antibody–drug conjugates were widely used to expose sufficient concentrations of chemotherapy agents to brain tumor cells for cancer treatment [7]. More importantly, nanomedicine emerged as one of the most promising and pivotal technologies in tumor diagnosis and therapy as it revived the application of toxic therapeutic agents such as doxorubicin for tumor treatment [8, 9]. Such nanocarriers mainly include liposome, polymeric micelle, nanoconjugate, gold nanoparticle, carbon nanotube, dendrimer, and phage-based nanoplatforms [10]. Among these nanoplatforms, polymeric nanoparticles, a kind of colloidal particle that consists of natural or synthetic polymers, have received the majority of attention due to their excellent stability and the ease of surface modification [11]. Besides, the particles, which are approximately 100 nm in size, are characterized by low clearance during circulation and a high enhanced permeability and retention (EPR) effect in solid tumor [12]. EPR effects, a phenomenon resulting from the defective architecture of tumor blood vessels and various vascular permeability factors, have a pivotal effect on the distribution and intratumoral penetration of nanoparticles [13]. It has been demonstrated that the microvascular pore size of a glioma is 7 to 100 nm; thus, the particles that are approximately 100 nm in diameter tend to be appropriate vehicles for penetration into the tumor interior through the large gaps between endothelial cells [14, 15].

Although EPR effect of tumor could facilitate the accumulation of chemotherapeutics-loaded nanoparticles into tumor parenchyma, using passive targeting seems inadequate, as splenic filtration and phagocytes in the liver may decrease the accumulation of nanoparticles in tumors [12]. Besides, the EPR effect of brain tumor was much weaker than that of peripheral tumors, indicating that BBB is still a main obstacle for brain tumor drug delivery [16, 17]. In this case, drug delivery system which could simultaneously targeted the BBB and brain tumor might facilitate drug penetrated from vascular and selectively accumulated in tumor cells [16, 17]. Advances in the analysis of tumor cell proteins have expanded the use of dual-targeted drug delivery. Among these, various receptors expressed on the surface of or inside tumor cells allow for active targeting delivery of agents into tumor tissues [18]. Receptor-mediated transcytosis could significantly increase the cellular association of chemotherapeutics [19]. Therefore, ligand-based dual targeting therapy might represent a new trend in improving the tumor extracting anti-cancer drugs through various kinds of receptors, including integrin alpha v beta 3, matrix metalloproteinase (MMP)-2, and low-density lipoprotein receptors (LDLR) [20–22].

As a predominant glycoprotein of Th2 lymphocytes and mast cells, interleukin-4 receptor (IL-4R) was reported to exist in high amounts in various tumor cells and endothelial cells [23–26]. In addition, the existing IL-4R was closely related to tumor genesis by numerous mechanisms [25]. For example, the polymorphisms in the interleukin (IL)-4/IL-13 pathway was reported previously to be associated with glioma [27]. Therefore, IL-4R-based tumor targeting drug delivery could be used for the treatment of several tumors. CRKRLDRNC peptide (AP1 peptide), selected through the phage-display technology, was reported to selectively bind to IL-4R [28]. Herein, we developed DOX-loaded nanoparticles and decorated on its surface with AP1 peptide for both glioma cells and angiogenesis dual targeting delivery of doxorubicin. The prepared nano-vehicle system could not only kill tumor cells directly, but also blocking the nutrient supplement which contributing to tumor progression at the same time through disrupted the vascular system of tumor tissue. In addition, the results of *in vivo* targeting experiments indicating that the AP1 peptide decorated nanoparticles not only could target tumor tissue but also have the penetrating ability from tumor vasculatures into tumor inner.

## RESULTS AND DISCUSSION

### Characterization of nanoparticles

**Figure 1:**
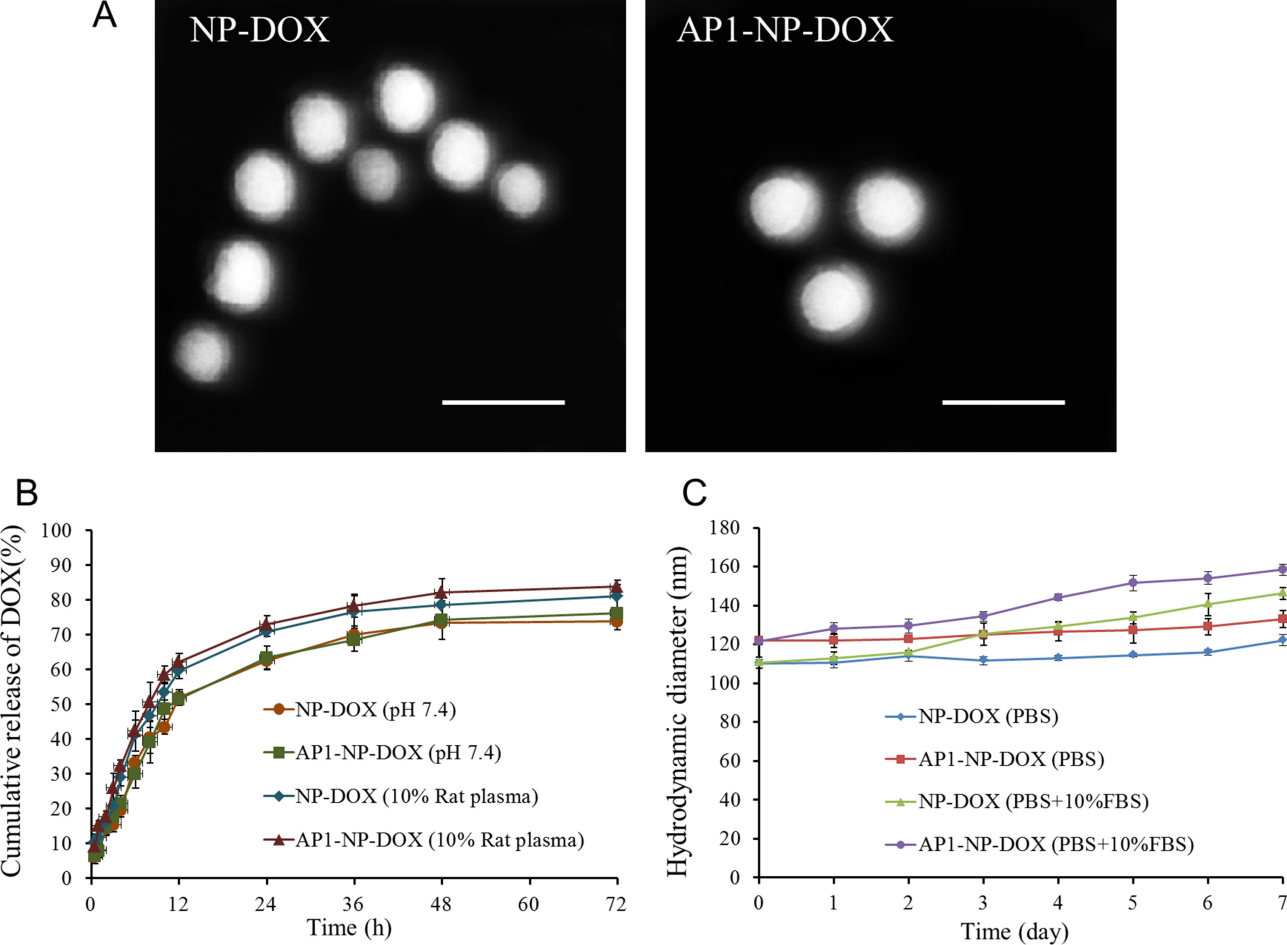
(**A**) The morphology of NP-DOX and AP1-NP-DOX photographed with the transmission electron microscope. The bar represents 200 nm. (**B**) Cumulative release (%) of doxorubicin from DOX formulations in different media. (**C**) Stability study of NP-DOX and AP1-NP-DOX in PBS containing or without 10% FBS.

The prepared DOX-loaded nanoparticles (NP-DOX) had a spherical shape and an average size of approximately 110 nm, as illustrated in Figure 1A. After functionalized with AP1 peptide, the nanoparticles (AP1-NP-DOX) had a negligible change in size and appearance (Figure 1A), with an average size of 120 nm. The zeta potential values of NP-DOX and AP1-NP-DOX were −29.7 mV and −26.3 mV, respectively. Drug loading capacity of DOX in NP-DOX and AP1-NP-DOX were about 1.43% and 1.37%, respectively, with the encapsulation efficiency of 56.33% (NP-DOX) and 53.74% (AP1-NP-DOX), respectively. These results indicated that peptide conjugation had no influence on the properties of nanoparticles.

For the *in vitro* drug release behavior, as shown in Figure 1B, both NP-DOX and AP1-NP-DOX showed a controlled-release pattern on the two conditions. And drug release from nanoparticles was slightly higher in the media containing plasma than that without plasma, which might be contributed to the enhanced matrix erosion in plasma [29].

As we know that an excellent stability of drug delivery system is of great importance to the therapy effect of chemotherapy. Therefore, the stability study of nanoparticles prepared in this study was performed. As shown in Figure 1C, results illustrated that the size of both nanoparticles did not change obviously within the determined days in the media of PBS, indicating that the prepared drug delivery system owned a well stability under such condition. However, a slightly size increase was observed for both nanoparticles when incubated with PBS containing 10% FBS, which was mainly contributed by the protein in FBS.

### Cellular uptake assay

**Figure 2:**
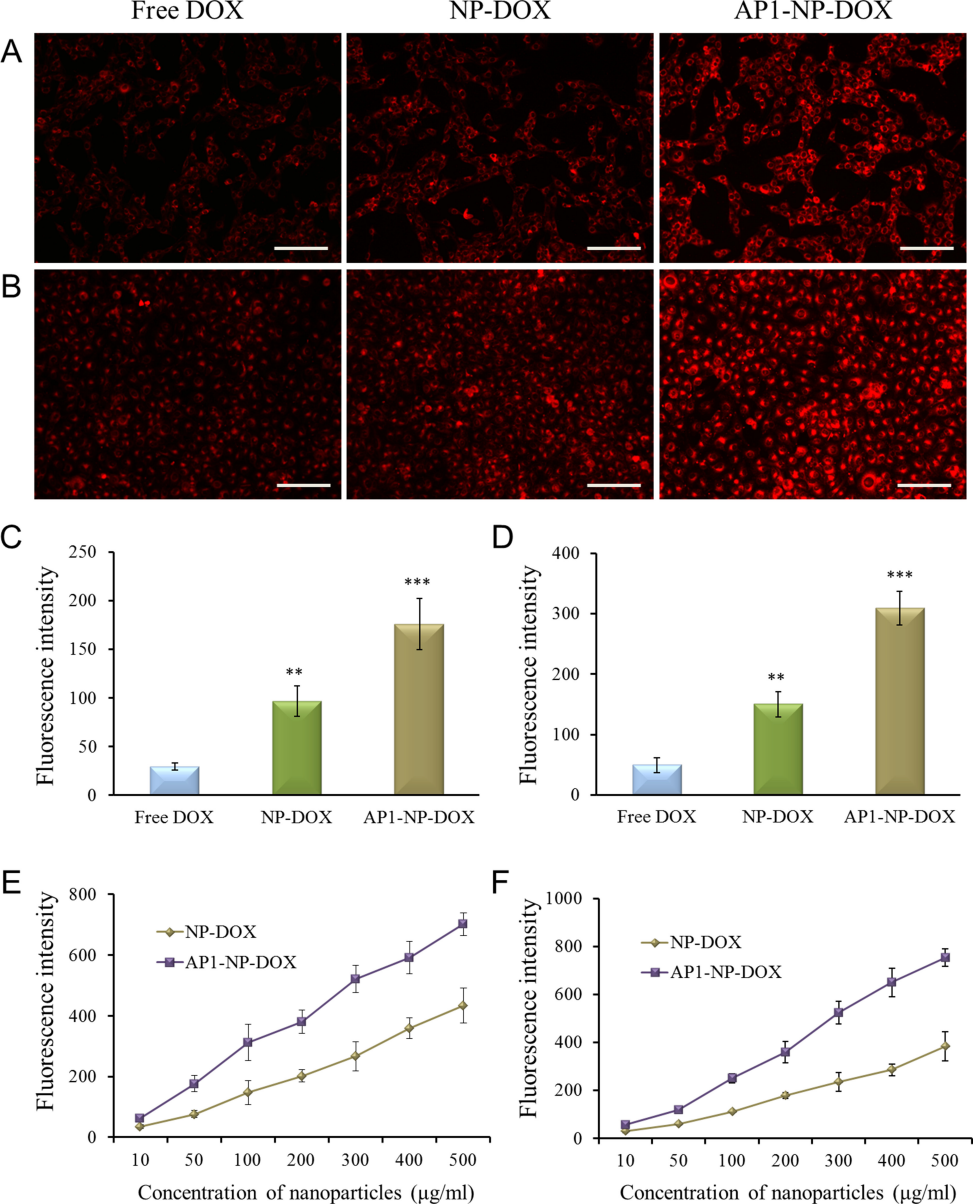
Cellular association of free DOX, NP-DOX, and AP1-NP-DOX, respectively (**A**) Uptake of free DOX and DOX-loaded nanoparticles in C6 cells after 3 h of incubation. (**B**) Uptake of free DOX and DOX-loaded nanoparticles in HUVEC cells after 3 h of incubation. (**C**) Quantitative analysis of cellular association of free DOX and DOX-loaded nanoparticles in C6 cells. (**D**) Quantitative analysis of cellular association of free DOX and DOX-loaded nanoparticles in HUVEC cells. (**E**) Uptake of NP-DOX and AP1-NP-DOX at various concentrations after 3 h incubation with C6 cells. (**F**) Uptake of NP-DOX and AP1-NP-DOX at various concentrations after 3 h incubation with HUVEC cells. Uptake was analyzed on the concentration of DOX. Data are presented as mean ± SD (**p < 0.01, ***p < 0.001, compared with cellular association of free DOX). The bar represents 100 μm.

To examine whether the peptide-modified nanoparticles could specifically accumulate in tumor cells and vascular endothelial cells, both cells were incubated with AP1-NP-DOX and NP-DOX at 37°C for 3 hours. As shown in Figure 2A and 2B, cellular uptake of AP1 peptide-functionalized nanoparticles was significantly higher than that of the unmodified ones. Moreover, the fluorescence intensity of both cells treated with DOX loaded nanoparticles was significantly stronger than that of free agents, indicating that nanoparticles could facilitate the cellular internalization of chemotherapeutics. The quantitative analysis further demonstrated this conclusion, as shown in Figure 2C and 2D. In addition, the quantitative determination under various concentrations of nanoparticles illustrated that the cellular uptake of nanoparticles was obviously concentration-dependent (Figure 2E and 2F).

### MTT assay

**Figure 3:**
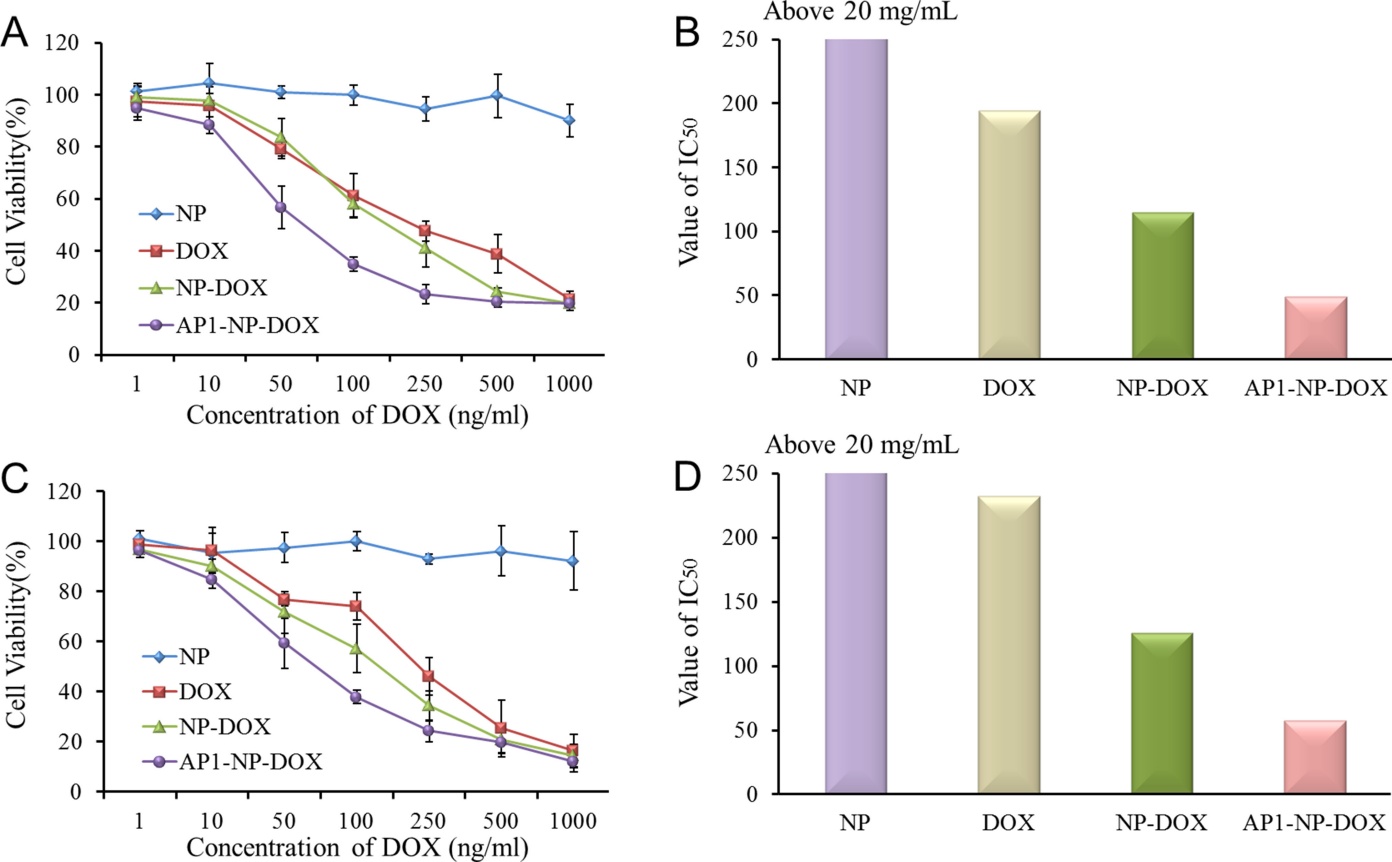
(**A**, **B**): Cell viability of C6 cells and HUVEC cells after being treated with serial concentrations of blank NP, free DOX, NP-DOX, and AP1-NP-DOX. (**C**, **D**): The value of IC_50_ of each formulation after incubation with C6 cells and HUVEC cells.

The MTT assay was used to evaluate the safety of polymer and antitumor activity of chemotherapeutics-loaded nanoparticles *in vitro*. The results, shown in Figure 3A–3D, indicated that AP1-NP-DOX resulted in lower cell viability when compared with NP-DOX. Besides, the cells treated with blank NP showed a negligible loss of cell viability. The IC50 value of each DOX formulation in C6 cells is 194.3 ng/mL (Free DOX), 114.8 ng/mL (NP-DOX), and 48.68 ng/mL (AP1-NP-DOX), respectively. In addition, the IC50 value of each DOX formulation in HUVEC cells is 232.2 ng/mL (Free DOX), 125.8 ng/mL (NP-DOX), and 57.49 ng/mL (AP1-NP-DOX), respectively. Importantly, the IC50 value of blank NP in C6 cells and HUVEC cells was more than 20 mg/mL. These results indicated that the polymer was safe to be used as a drug carrier and AP1 peptide could significantly facilitate the antitumor efficacy of DOX-loaded nanoparticles.

### Tumor targeting assay

**Figure 4:**
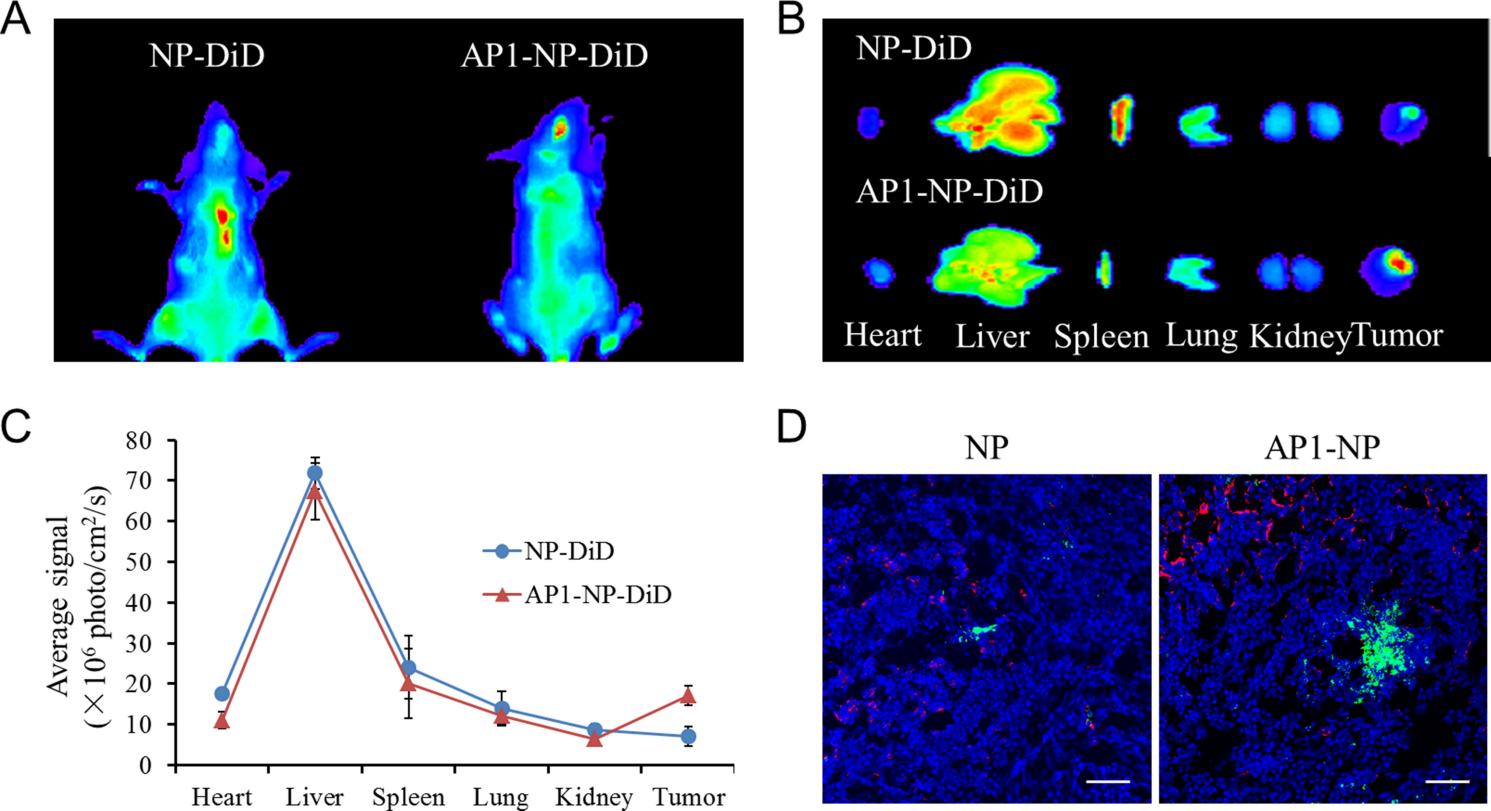
(**A**) *In vivo* imaging of tumor-bearing mice 24 h after injection with NP-DiD or AP1-NP-DiD. (**B**) *Ex-vivo* imaging of tumor tissues and organs (include heart, liver, spleen, lung, kidney) 24 h after injection with NP-DiD or AP1-NP-DiD. (**C**) Semi-quantitative analysis of fluorescence intensity of NP-DiD and AP1-NP-DiD in tumor sites and organs. (**D**) *In vivo* distribution of nanoparticles in glioma at 3 h after administration. Blue: cell nuclei stained by DAPI. Green: Courmarine-6-labeled nanoparticles. Red: CD31 antibody labeled microvessels. The bar represents 100 μm.

To investigate the tumor targeting ability of AP1-functionalized nanoparticles *in vivo*, tumor-bearing mice were established and the tumor targeting assay was then performed as reported previously [30]. As shown in Figure 4A, the mice treated with NP-DiD exhibit only a little accumulation of nanoparticles in tumor site. Nevertheless, the mice given AP1-NP-DiD showed much stronger fluorescence intensity in the brain tumor site. Furthermore, semi-quantitative analysis of fluorescence intensity of brain tumors demonstrated that the amount of AP1-NP-DiD accumulated in tumor was approximately three times higher than that of NP-DiD (Figure 4C). The *ex vivo* imaging was further performed at 24 hours after administration of NP-DiD or AP1-NP-DiD. Results in Figure 4B and Figure 4C indicated that AP1 modified nanoparticles could selectively accumulated in tumor tissue, and then leading to a less distribution in normal tissues than the unmodified ones. These results together illustrated that AP1-NP-DiD had a superior *in vivo* tumor targeting ability than NP-DiD.

### Brain uptake of nanoparticles

**Figure 5:**
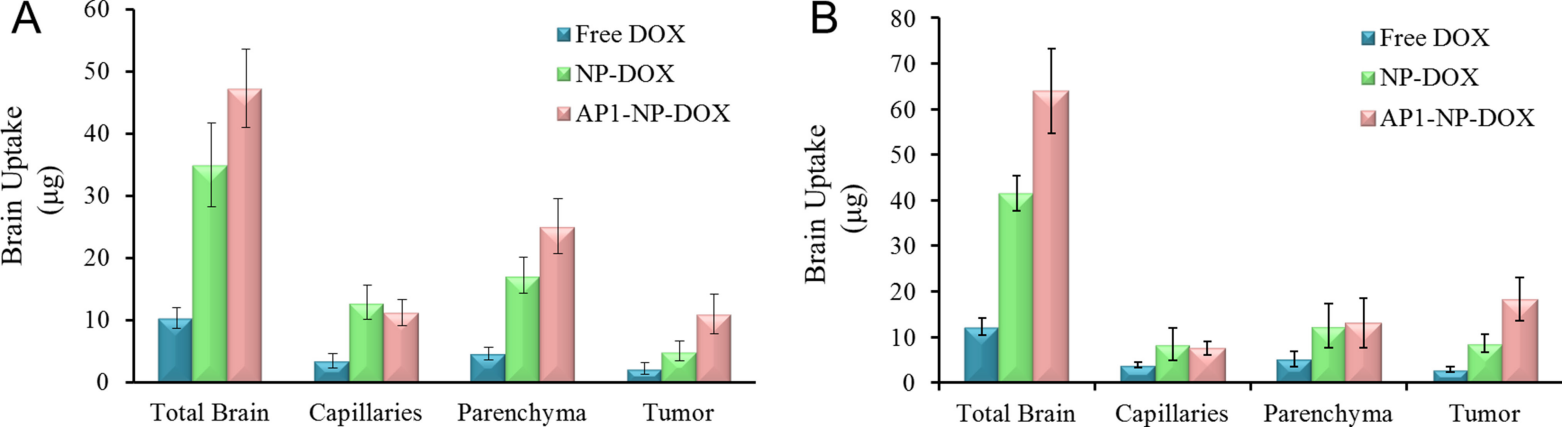
Brain uptake of different formulations 3 h (**A**) and 6 h (**B**) after intravenous injection with free DOX, NP-DOX, and AP1-NP-DOX, respectively. The brain capillary depletion was performed. The amount of formulations in total brain, capillaries, parenchyma and tumors were evaluated.

To quantify nanoparticles in the brain tumors, DOX-loaded nanoparticles were developed and administered to the tumor-bearing mice. Three hour or six hours later, the right hemisphere of the brain, which was implanted with tumors, was isolated on ice-bath and treated with capillary depletion. In addition, nanoparticles or free DOX in the left hemisphere of the brain was monitored to evaluate the distribution of free DOX, NP-DOX, and AP1-NP-DOX in total brain. Results shown in Figure 5 demonstrate that AP1-NP-DOX accumulated the most in total brain. Furthermore, AP1-NP-DOX exhibited a significant increase in tumor permeability compared to NP-DOX and free DOX, demonstrating the great tumor targeting ability of AP1 peptide. However, the distribution of AP1-NP-DOX in parenchyma was much higher than that of NP, which was possibly caused by the immediate diffusion of AP1-NP-DOX in brain tissue. As shown in Figure 5B, 6 hours after i.v injection of AP1-NP-DOX, the proportion of tumor accumulation of AP1-NP-DOX was further enhanced and the distribution of AP1-NP-DOX in parenchyma was significantly decreased. These results together suggest that the AP1 peptide can improve the brain and tumor penetration of the DOX-loaded nanoparticles to a large extent.

### *In vivo* distribution of nanoparticles in tumor tissue

An adequate tumor tissue accumulation and deep penetration of chemotherapeutics are the important prerequisites for the treatment of cancer. In this study, the AP1 peptide was characterized with highly tumor affinity and modified on the surface of DOX-loaded nanoparticles for glioma targeting therapy. To evaluate whether the nanoparticles could target the angiogenesis and then access the inside avascular region, the fluorescence immunoassay was performed. As shown in Figure 4D, the fluorescence intensity of NP in tumor slides showed weaker signals than that of AP1-NP. Besides, only a small amount of unmodified nanoparticles gathered around the tumor blood vessels, but much more AP1-functionalized nanoparticles aggregated around vascular and further penetrated into tumor parenchyma. These results indicate that the tumor targeting drug delivery system is a superior strategy for malignant tumor therapy.

### Survival of mice after treatment with different formulations

**Figure 6:**
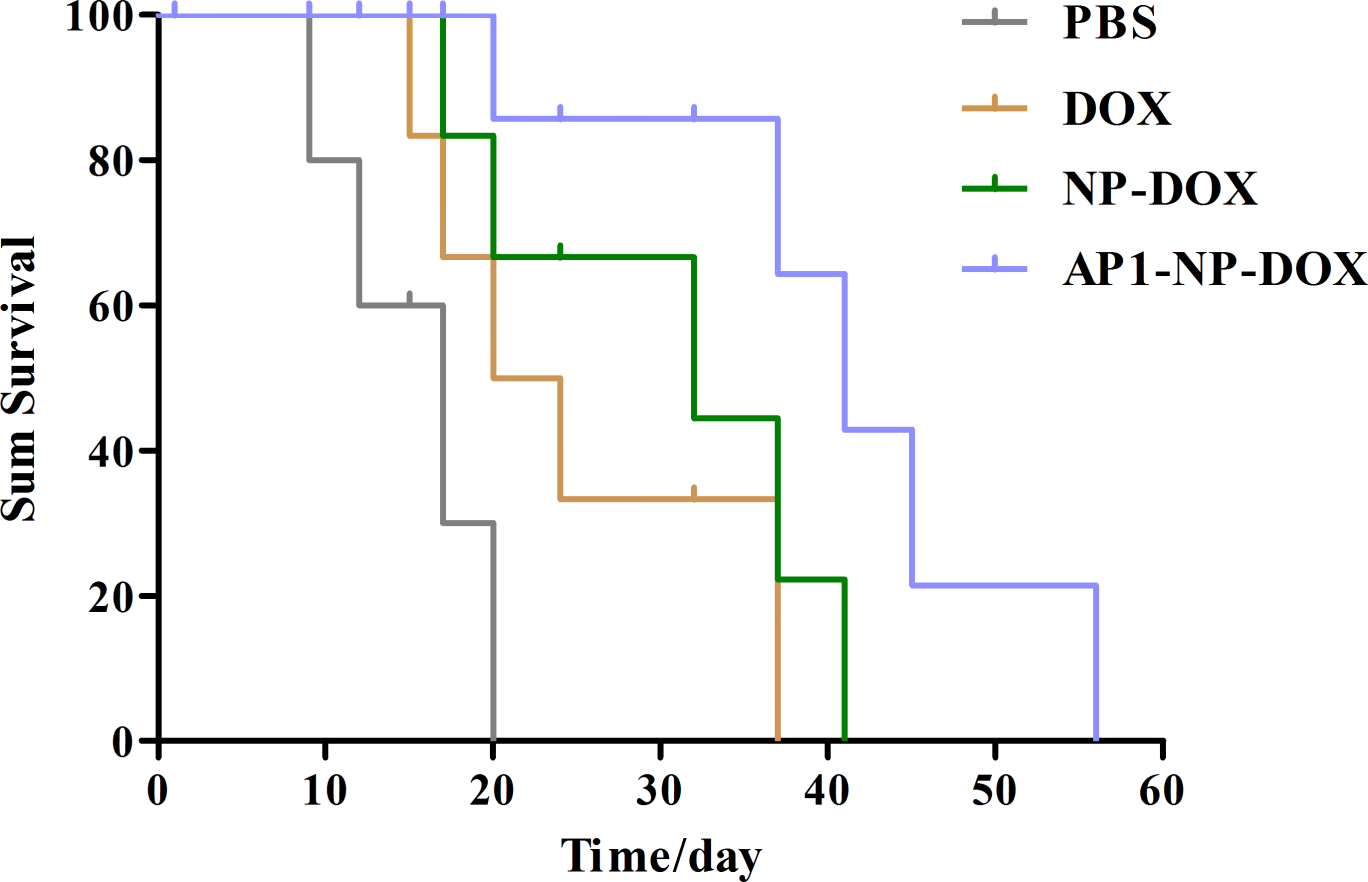
Survival curve of the brain glioma-bearing mice treated with different formulations at the same dose of DOX (10mg/kg); *n* = 7

On the basis of the super tumor targeting ability of AP1 peptide, it would be expected that AP1-NP-DOX could result in a survival advantage when compared with the others [NP-DOX, free DOX, and phosphate-buffered saline (PBS)]. Consistently, results demonstrated the expectation well, with the mice treated with AP1-NP-DOX achieving the longest medium survival time (47 days) (Figure 6). Moreover, the medium survival time of NP-DOX treated mice was 35 days, significantly longer than that of PBS (20 days) and free DOX (17 days), which was attributed primarily to the EPR effect.

### Cell apoptosis of tumor sections

**Figure 7:**
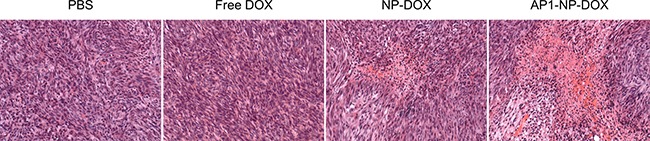
The histopathologic changes in glioma after treatment with various DOX formulations was measured by H&E staining The PBS group was used as control.

Hematoxylin and eosin (H&E) staining was introduced to evaluate the toxicity of DOX formulations to glioma cells *in vivo* with PBS as the control. As shown in Figure 7, NP-DOX induced a slightly higher apoptosis of tumor cells than free DOX, while AP1-NP-DOX induced a much higher apoptosis of tumor cells than NP-DOX. These results indicated that EPR effect could facilitate accumulation of nanoparticles other than small molecules in tumor sites. Besides, much more nanoparticles could accumulate in tumor tissue after functionalized with AP1 peptide.

### Inhibition of tumor growth after treated with DOX formulations

**Figure 8:**
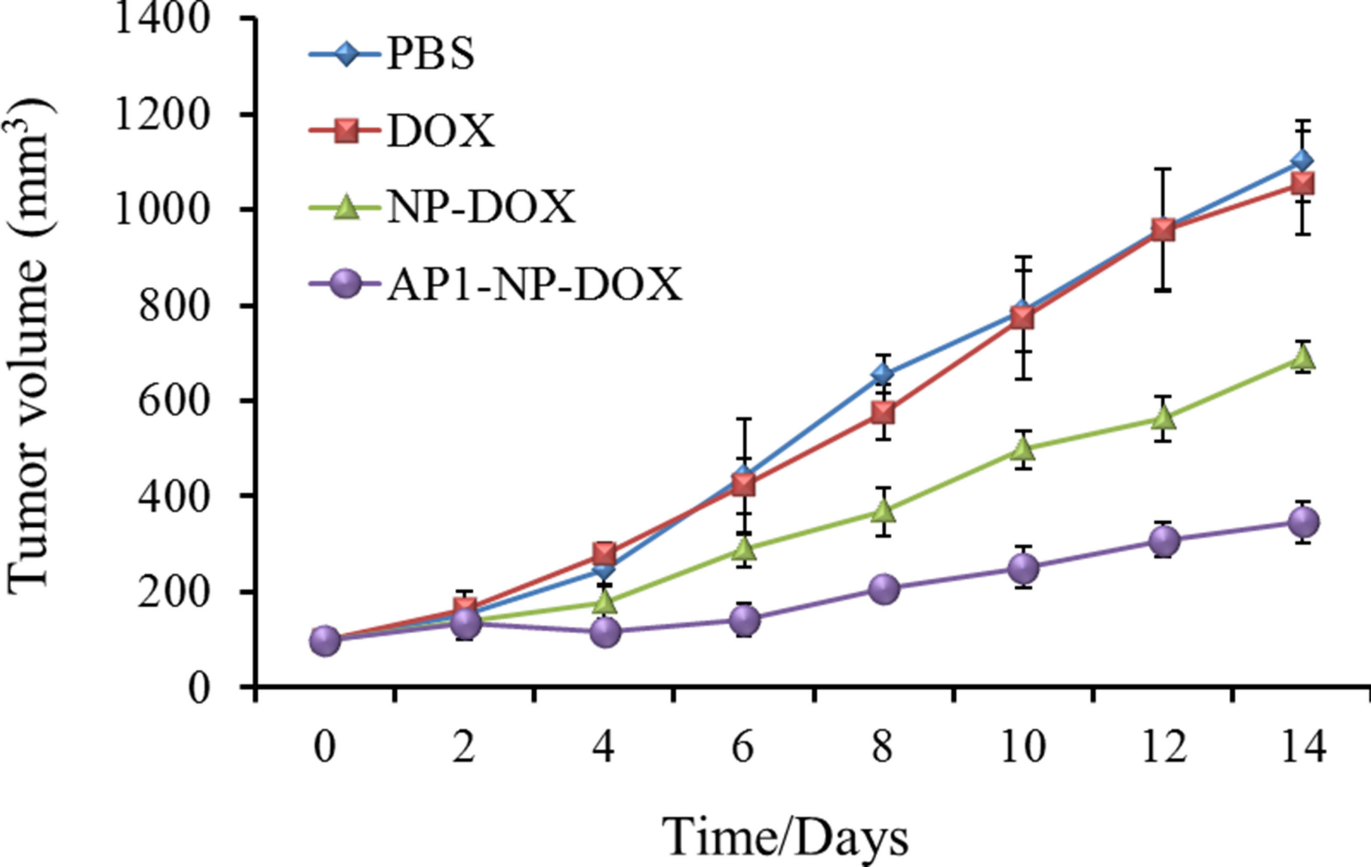
Tumor inhibition after treated with PBS, free DOX, NP-DOX and AP1-NP-DOX at the DOX dose of 10 mg/kg *n* = 6

The evaluation of anti-tumor efficacy of various DOX formulations in subcutaneous xenograft tumors has been performed. As shown in Figure 8, the group treated with AP1-NP-DOX exhibited the best anti-tumor efficacy, demonstrating that AP1 peptide could contribute to the accumulation of nanoparticles into tumor sites. Furthermore, when compared with PBS and free DOX, mice given with NP-DOX showed a superior tumor inhibition rate due to the EPR effect of the tumor microenvironment.

## MATERIALS AND METHODS

### Materials

AP1 peptide was purchased from Sangon Biotech Co. (Shanghai; HPLC-purified; purity > 95%, identified by MS). Sodium cholate was from Shanghai Chemical Reagent Company. Methoxy-poly (ethylene glycol)-poly (lactic acid) (MPEGePLA, Mw 33,000 Da) and R-carboxyl-poly (ethylene glycol)-poly (lactic acid) (COOH-PEG-PLA, Mw 33,400 Da) block copolymers were purchased from Adamas Corporation (Shanghai local agent, China). Doxorubicin (DOX) was obtained from Beijing Huafeng United Technology (China). DiD (1,1′-dioctadecyl-3,3,3′,3′-tetramethy-lindotricarbocyanine iodide), a near-infrared dye used for the evaluation of *in vivo* targeting assay, was obtained from Biotium (Invitrogen, USA). Alexa Fluor 594 antimouse CD31 antibody was purchased from Biolegend. Dulbecco's modified Eagle medium (DMEM; high glucose) cell culture medium, certified fetal bovine serum (FBS), penicillin/streptomycin stock solutions, and 0.25% Trypsin-EDTA were purchased from Gibco (USA). All other chemicals, if not indicated specifically, were obtained from Sigma-Aldrich.

### Cell culture

The primary human umbilical vein endothelial cells (HUVEC cells) were purchased from Cascade Biologics (Portland, OR, USA), and the C6 glioma cell line was obtained from the American Type Culture Collection. Both cells were cultured in DMEM containing 10% FBS, 2 mM glutamine, 100 U/mL penicillin, and 100 mg/mL streptomycin and maintained in a standard condition with a humidified atmosphere of 5% CO_2_ and temperature of 37°C.

### Animals

Nude mice were obtained from the Sino-British Sippr/BK Lab Animal Ltd. (China). Mice were maintained in a pathogen-free environment and handled in accordance with guidelines from the Shanghai Tenth People's Hospital Affiliated with Tongji University.

### Preparation of nanoparticles

DOX-loaded nanoparticles (NP-DOX) were prepared by the emulsion-solvent evaporation method [31]. The blend of 30 mg MPEG-PLA and 5 mg HOOC-PEG-PLA was dissolved by the dichloromethane. Thereafter, 1 mg DOX was added followed by the addition of 2 mL of 0.8% sodium cholate aqueous solution. Then, a probe sonicator (Scientz Biotechnology Co. Ltd., China) was applied to form the DOX-loaded nanoparticles. After ultrasonication at 8 s for 20 times (220 W) in an ice bath, dichloromethane was evaporated using a ZX-98 rotary evaporator (Shanghai Institute of Organic Chemistry, China). Finally, the nanoparticle-contained solutions were centrifuged using a TJ-25 centrifuge (Beckman Counter, USA) to collect the DOX-loaded nanoparticles. The AP1 peptide functionalized nanoparticles (AP1-NP-DOX) were prepared through an EDC/NHS technique [32]. Briefly, the collected nanoparticles were resuspended by deionized water followed by the addition of excess EDC (200 mM) and NHS (100 mM) and then were mildly stirred for 45 min at room temperature. The prepared N-hydroxysuccinimide-activated nanoparticles were concentrated by centrifugation. To develop the peptide decorated nanoparticles, the activated NP-DOX was incubated with 170 μg AP1 peptide for 4 h. Finally, the functionalized nanoparticles were obtained by centrifugation at 14,500 rpm for 45 min.

### Characterization of nanoparticles

The average grain diameter of nanoparticles and zeta potential were determined by a dynamic light scattering detector (Zetasizer, Nano-ZS, Malvern, UK). To observe the morphology of nanoparticles, both NP-DOX and AP1-NP-DOX were negatively stained with sodium phosphotungstic solution. Finally, the nanoparticles were detected under a transmission electron microscope (TEM; H-600, Hitachi, Japan). Besides, the drug encapsulation efficiency (EE) and loading capacity (LC) were determined using the fluorescence spectrometry (Ex: 480 nm and Em: 580 nm).

The release of loaded chemotherapeutics from nanoparticles was examined *in vitro* via an equilibrium dialysis method with the phosphate buffer solution (PBS, pH7.4) containing or without 10% (v/v) rat plasma as the release media [33]. Briefly, one milliliter of DOX formulation containing 100 μg/mL of DOX was introduced into a dialysis bag (MWCO 1 kDa; Green Bird Inc., Shanghai, China) containing 30 mL of release media. Then the dialysis bag was incubated in a shaker (100 rpm) at 37°C for 72 h and 0.2 mL of release sample was withdrawn at the predetermined time points. The concentration of DOX was analyzed using a fluorescence spectrometry.

The stability of DOX-loaded nanoparticles (NP-DOX and AP1-NP-PDOX) was determined through using PBS (pH7.4) containing or without 10% (v/v) fetal calf serum (FBS) as the media. Briefly, 10 mg of NP-DOX or AP1-NP-DOX were suspended in PBS and PBS containing 10% (v/v) FBS, respectively, and then incubated for seven days followed by examined the changes of the nanoparticles size every day.

### Cell uptake

HUVEC cells were selected as the representative cells to evaluate the angiogenesis targeting ability of various formulations. C6 cells were used to investigate the tumor cell targeting ability of nanoparticles or chemotherapeutics. Both cells were seeded in 12-well plates with a density of 1 × 10^5^ cells per well. After 24 h, the cells were treated with free DOX, NP-DOX, and AP1-NP-DOX at the DOX concentration of 20 ng/mL. Three hours later, the cells were rinsed by PBS to remove the unassociated nanoparticles or free DOX, and then fixed with 4% paraformaldehyde for 10 min. Finally, the cells were subjected to qualitative analysis under fluorescence microscopy (Leica, Germany). For quantitative evaluation, the cells were digested by trypsin after being washed with PBS. The collected cells were subjected to analysis with a flow cytometry system (BD, USA). The cells treated with various concentrations of nanoparticles were further analyzed to study the manner of uptake.

### MTT assay

MTT assay was performed to evaluate the cytotoxicity of free DOX, blank NP, NP-DOX, and AP1-NP-DOX. Both C6 cells and HUVEC cells (1 × 10^4^ cells/100 μL per well) were seeded in 96-well plates and cultured at 37°C in 5% CO_2_. Solutions containing various formulations were diluted by culture medium, with the final concentration ranging from 0.001 to 1 μg/mL. After incubation with the aqueous solution for 48 h, 10 μL of MTT (5 mg/mL) dissolved by the PBS was added to the cells in each well of plates. Four hours later, the cells were lysed with 50% N, N-dimethylformamide containing 20% sodium dodecyl sulfate and measured at 570 nm by a SpectraMax M5 (Molecular Devices, Sunnyvale, CA, USA).

### Established the intracranial model of glioma

The evaluation of the uptake of peptide mediated nanoparticles in an animal model of glioma was performed by C6 cells. In brief, nude mice were deeply anesthetized by isofluraneq. The scalp was swabbed with alcohol and the skin was cut. Thereafter, 5 μL of cell suspension (1 × 10^4^ cells/mL), prepared with PBS and kept on ice until injection, was injected into the corpus striatum of the right hemisphere with a 10-μL syringe. Finally, the skin was sutured with three knots, and the mice were raised under standard conditions. Further brain tumor assays were performed at 10 days post tumor implantation.

### Tumor targeting assay

The tumor-bearing mice were randomly divided into two groups (three mice per group). For the *in vivo* and *ex vivo* imaging, NP-DiD and AP1-NP-DiD were prepared in the same way as NP-DOX and AP1-NP-DOX and intravenously injected into mice at a DiD dose of 2 mg/kg. Subsequently, mice were anesthetized with chloral hydrate and imaged using the IVI^®^ Spectrum system (Caliper, Hopkington, MA, USA) at 24 hours. The mice were euthanized after heart perfusion with saline and 4% paraformaldehyde and the *in vivo* imaging. Then, the tumors and organs (including heart, liver, spleen, lung, and kidney) were collected and imaged using the IVI^®^ Spectrum system.

### Brain uptake of nanoparticles

To quantify the distribution of chemotherapeutics-loaded nanoparticles and free DOX in brain tissue, the tumor-bearing mice were randomly divided into 3 groups (*n* = 3) and administered with free DOX, NP-DOX, and AP1-NP-DOX (at the dose of 5 mg/kg DOX in solution), respectively. Three hours or six hours after injection, the mice were euthanized and their brains were surgically removed and dissected. Then, the collected brains were maintained in cold PBS containing protease inhibitors until processing. To determining the levels of DOX in tumor-bearing brain, both the implanted hemisphere (right hemisphere) and the non-implanted hemisphere (left hemisphere) were incubated with 2 mg/mL collagenase and 0.125% trypsinogen at 37°C for 30 min. Then the samples were homogenized on ice with 3-fold volumes of deionized water followed by incubated with 2 mL of chloroform/methanol (4:1, v/v) and vortexed for 2 min afterwards. After that, the subnatant was collected by centrifugating the suspension at 8000 rpm for 10 min and evaporated to remove the organic solvent. Finally, the residue was dissolved with 200 μL of methanol and filtered using a 200-mesh screen. Then the suspension was analyzed using flow cytometry (BD Biosciences, USA).

### Distribution of nanoparticles in glioma

To investigate the distribution of nanoparticles in glioma tissue, 6 glioma *in situ*-bearing mice were randomly divided into two groups (*n* = 3) and intravenously injected with coumarin-6-labeled NP and AP1-NP, respectively. After 3 h, the mice were sacrificed with the brain slides prepared as described previously [30]. For analyzing the localization of nanoparticles at the tumor site, Alexa Fluor 594 antimouse CD31 antibody was introduced to stain the tumor neovascular and then all of the slides were subjected to confocal microscopy analysis (LSM710, Leica) after staining the nucleus with DAPI.

### Treatment of brain tumor-bearing mice *in vivo*

To study the anti-tumor effect of various formulations, the tumor-implanted mice (glioma *in situ*) were randomly divided into 4 groups (*n = 7*) and treated with free DOX, NP-DOX, and AP1-NP-DOX, respectively. Mice given PBS served as the control group. The dosage of DOX was 10 mg/kg. Finally, the median survival time of each mouse was carefully recorded and calculated using a two-tailed Wilcoxon's signed-rank test.

To evaluate the cell apoptosis induced by DOX-loaded nanoparticles prepared in this study, glioma-bearing mice were introduced and treated with free DOX, NP-DOX and AP1-NP-DOX (dosing at 10 mg/kg), respectively, at 0, 2, 4, and 6 days (*n* = 3). After treatment, all the mice were sacrificed, and the tumor-bearing brains were harvested and subjected to H&E staining.

### Inhibition of tumor growth in the subcutaneous xenograft tumor model

To further evaluate the anti-tumor activity of various DOX formulations, a subcutaneously C6 xenograft tumor model was established as reported previously [34]. Briefly, 1 × 10^5^ C6 cells were subcutaneously injected into the selected flanks of nude mice. Then, the mice were raised in standard conditions and supplied with enough food and water. Following the Gompertzian kinetics of solid tumor, the mice with tumors reaching ~100 mm^3^ in volume were used [35].

To evaluate the anti-tumor efficacy of different formulations in subcutaneous xenograft tumors, free DOX, NP-DOX and AP1-NP-DOX (the dosage of DOX was 10 mg/kg) were administered to tumor-bearing mice through the tail vein, and the mice treated with PBS were used as the negative control group. The tumor volumes [Volume = 0.5 × length × (width)^2^] were observed carefully and recorded in the next 14 days.

### Statistical analysis

Data are expressed as mean ± standard deviation (SD). Statistical analyses were performed using student's *t* test when one group was compared with the control group. Analysis of variance (ANOVA) was used when two or more groups were compared. For survival studies, statistical analysis was performed using a two-tailed Wilcoxon's signed-rank test. *P* < 0.05 was considered to be statistically significant.

## CONCLUSIONS

IL-4R has been demonstrated previously to be highly expressed on tumor cells and angiogenesis. Therefore, IL-4R-based active targeting drug delivery could be applied for efficient glioma treatment through the receptor-mediated transcytosis [36]. In the present study, polylactic acid-based nanoparticles were developed and loaded with DOX for the treatment of glioma. To make the nanoparticle selectively accumulate into tumor tissues, we decorated its surface with a high affinity tumor peptide, AP1, which selectively binds to IL-4R. *In vitro* cellular experiments demonstrated a superb dual targeting ability to tumor cells and angiogenesis for AP1 peptide as AP1-decorated nanoparticles showed a higher cellular internalization compared with the unmodified ones. Besides, both C6 cells and HUVEC cells treated with AP1-NP-DOX achieved the lowest cellular viability. Furthermore, *in vivo* tumor targeting assay showed that AP1-NP-DOX exhibited the strongest fluorescence intensity and results of tumor uptake experiments indicating a potential ability of BBB crossing for AP1 peptide. The superior dual targeting performance of AP1-NP-DOX resulted in an effective glioma inhibition as demonstrated in the anti-glioma study. These results together indicated that DOX-loaded dual targeting nanoparticles prepared in this study could be used for the therapy of glioma.
